# MEK inhibitors induce Akt activation and drug resistance by suppressing negative feedback ERK‐mediated HER2 phosphorylation at Thr701

**DOI:** 10.1002/1878-0261.12102

**Published:** 2017-07-19

**Authors:** Chia‐Hung Chen, Te‐Chun Hsia, Ming‐Hsin Yeh, Tsung‐Wei Chen, Yun‐Ju Chen, Jung‐Tsu Chen, Ya‐Ling Wei, Chih‐Yen Tu, Wei‐Chien Huang

**Affiliations:** ^1^ Graduate Institute of Clinical Medical Science China Medical University Taichung Taiwan; ^2^ Division of Pulmonary and Critical Care Medicine Department of Internal Medicine China Medical University Hospital Taichung Taiwan; ^3^ Department of Respiratory Therapy China Medical University Taichung Taiwan; ^4^ Department of Internal Medicine Hyperbaric Oxygen Therapy Center China Medical University Hospital Taichung Taiwan; ^5^ Department of Surgery Chang Shan Medical University Taichung Taiwan; ^6^ Department of Pathology China Medical University Hospital Taichung Taiwan; ^7^ Department of Pathology Tainan Municipal An‐Nan hospital China Medical University Taichung Taiwan; ^8^ Department of Medical Research E‐Da Hospital Kaohsiung Taiwan; ^9^ Department of Biological Science & Technology I‐Shou University Kaohsiung Taiwan; ^10^ School of Medicine I‐Shou University Kaohsiung Taiwan; ^11^ Graduate Institute of Clinical Dentistry National Taiwan University Taipei Taiwan; ^12^ Department of Dentistry National Taiwan University Hospital Taipei Taiwan; ^13^ Center for Molecular Medicine China Medical University and Hospital Taichung Taiwan; ^14^ School of Medicine China Medical University Taichung Taiwan; ^15^ Department of Life Science National Chung Hsing University Taichung Taiwan; ^16^ Graduate Institute of Biomedical Sciences China Medical University Taichung Taiwan; ^17^ The Ph.D. program for Cancer Biology and Drug Discovery China Medical University Taichung Taiwan; ^18^ Department of Biotechnology Asia University Taichung Taiwan

**Keywords:** Akt, clathrin, HER2, MEK, resistance

## Abstract

Targeting the MEK/ERK pathway has been viewed as a promising strategy for cancer therapy. However, MEK inhibition leads to the compensatory PI3K/AKT activation and thus contributes to the desensitization of cancer cells to MEK inhibitors. The underlying molecular mechanism of this event is not yet understood. In this study, our data showed that the induction of Akt activity by MEK inhibitors was specifically observed in HER2‐positive breast cancer cells. Silence of HER2, or overexpression of HER2 kinase‐dead mutant, prevents the induction of Akt activation in response to MEK inhibition, indicating HER2 as a critical regulator for this event. Furthermore, HER2 Thr701 was demonstrated as a direct phosphorylation target of ERK1/2. Inhibition of this specific phosphorylation prolonged the dimerization of HER2 with EGFR in a clathrin‐dependent manner, leading to the enhanced activation of HER2 and EGFR tyrosine kinase and their downstream Akt pathway. These results suggest that suppression of ERK‐mediated HER2 Thr701 phosphorylation contributes to MEK inhibitor‐induced Akt activation.

**Abbreviations**
CCP: clathrin‐coated pits
CME: clathrin‐mediated endocytosis
DC: dansylcadaverine
KD: kinase‐dead
mTORC1: mammalian target of rapamycin complex 1
PI3K: phosphatidylinositol 3‐kinase
PLA: proximity ligation assay
RTK: receptor tyrosine kinase

## 1 Introduction

In response to the activation of receptor tyrosine kinase (RTK), ERK1/2 are triggered through RAS/RAF/MEK cascade to regulate cell proliferation, differentiation, survival, and cell motility (Chang and Karin, 2001). Activating mutations in these MAPK‐related genes were found in many cancer types, such as breast cancer, melanoma, mesothelioma, and non‐small‐cell lung cancer, to drive the aberrant activation of ERK1/2 signaling (Davies *et al*., 2002; Giltnane and Balko, 2014; Ji *et al*., 2007; Kempf *et al*., 2016; Millington, 2013). The deregulation of MAPK pathway represents some of the driving characteristics and key hallmarks of the cancer cell (Hanahan and Weinberg, 2000, 2011). Consequently, many cancers bearing these MAPK‐related mutant genes exhibit a survival addiction to ERK1/2 signaling (Caunt *et al*., 2015; Kidger and Keyse, 2016). RAF and MEK inhibitors therefore are being developed as treatments for cancers with activated RAF/MEK/ERK signaling.

More than half of melanomas harbor activating V600E mutation of BRAF (BRAFV600E) (Long *et al*., 2012), a critical driver gene for the proliferation and survival of melanoma cells through the activation of MAPK pathway (Dhomen and Marais, 2009; Fecher *et al*., 2008). MEK inhibitors, such as AZD6244 (Selumetinib^®^), have been approved for uveal melanoma by FDA. In preclinical studies, MEK inhibitors were also shown to reduce the lung metastasis of breast cancer and inhibit breast cancer stem cells, suggesting the clinical potential for patients with breast cancer whose tumors are MAPK dependent (Bartholomeusz *et al*., 2015). However, with the exception of BRAF‐mutant melanomas, the clinical therapeutic efficacy of MEK inhibitors as single agent for other cancer types has been underwhelming.

Among several potential mechanisms underlying the lack of efficiency of MEK inhibitors, feedback activation of PI3K/AKT pathway has been invoked (Mirzoeva *et al*., 2009; Turke *et al*., 2012). Under the control of RTKs, MAPK pathway and phosphatidylinositol 3‐kinase (PI3K)/Akt pathway coordinately regulate mammalian target of rapamycin complex 1 (mTORC1) activity and other effectors involved in cell survival, metabolism, proliferation, and metastasis (Corcoran *et al*., 2011). The interplays between these two oncogenic pathways potentially impact the sensitivity of cancers to kinase inhibitors. Treatment with MEK inhibitors also led to an increase in AKT activity, reducing the cellular sensitivity of breast and gastric cancer cells to these inhibitors (Hoeflich *et al*., 2009; Mirzoeva *et al*., 2009; Yoon *et al*., 2009). The compensatory activation of PI3K/Akt pathway determines the susceptibility of cancer cells to MEK inhibition. However, the underlying mechanisms of the compensatory Akt activation by MEK inhibition are still not clear.

In this study, our data showed that ERK phosphorylates HER2 at Thr701 and that suppression of this phosphorylation by MEK inhibitors enhances the protein interaction between clathrin and HER2 to mediate the dimerization of HER2/EGFR complex to drive Akt signaling. Our findings provide greater details of the molecular mechanisms by which MEK inhibition induces the compensatory Akt activation in conferring drug resistance.

## 2 Materials and methods

### 2.1 Cell culture

Human HER2‐positive breast cancer cell lines (SKBR3, BT474, MCF7/HER2) and HER2‐negative breast cancer cell lines (MDA‐MB‐231, MDA‐MB‐468, MCF7) were obtained from ATCC (Manassas, VA, USA). All cell lines within 15 passages were cultured in Dulbecco's modified Eagle's medium/F12 supplemented with 10% fetal bovine serum, 100 U·mL^−1^ penicillin, and 100 μg·mL^−1^ streptomycin at 37 °C in a humidified atmosphere of 95% air and 5% CO_2_.

### 2.2 Plasmid and siRNA transfection

Cancer cells were transfected with plasmid or siRNA by using Lipo‐Fectamine 2000 (Invitrogen, Waltham, MA, USA), TransIT‐2020 or TransIT‐X2 (Mirus, Madison, WI, USA) transfection reagent according to the manufacturer's instruction. After 48–72 h of transfection, cells lysates were harvested and subjected to further analysis.

### 2.3 Western blot analyses

Total cell lysates were extracted with RIPA buffer (50 mm Tris, 150 mm NaCl, 10 mm EDTA, 1% Triton X‐100, 0.1% SDS) plus complement and protease inhibitors. The concentration of protein was measured by Bradford protein assay (Bio‐Rad protein assay). Equal amounts of proteins were separated on SDS/PAGE and transferred to PVDF membranes. After incubation with 5% milk for 1 h at room temperature, the membranes were blotted with indicated antibodies at 4 °C overnight followed by incubation with HRP‐labeled secondary antibody for 1 h. After wash with TBST buffer for three times, the chemiluminescence signal was catalyzed with ECL (GE Healthcare, Chicago, IL, USA or Millipore, Billerica, MA, USA) for detecting the level of protein expression. Antibodies against pHER2 Y1221/1222 (#2243), pEGFR Y1068 (#2236), pHER3 Y1289 (#4791), pAKT S473 (#9271), pAKT T308 (#9275), AKT (#9272), pERK (#9106), ERK (#9102), and myc‐tag (#2278) were purchased from Cell Signaling (Danvers, MA, USA). Antibodies against HER2/Neu (sc‐284), EGFR (sc‐03), HER3 (sc‐285), clathrin (sc‐12734), and HA probe (sc‐805) were purchased from Santa Cruz (Dallas, TX, USA). The levels of protein expression and phosphorylation were analyzed by western blot, and the folds of increase in Akt phosphorylation were quantified by using image j software (NIH, Bethesda, MD, USA) and normalized with total protein level of Akt. Some selected raw data was shown in the Fig. S3.

### 2.4 Immunoprecipitation

After indicated treatment, cancer cells were trypsinized and incubated in 2% formaldehyde for 7 min at room temperature with gentle rotation. Cross‐linking reaction was stopped by adding glycine/PBS at a final concentration of 1.25 m for 3 min at room temperature with gentle rotation. Total cell lysate was prepared with RIPA buffer, and 1 mg cell lysate was adjusted to 1 mL of reaction volume with IP buffer (20 mm Hepes, 100 μm KCl, 2 mm MgCl_2_, 15 mm NaCl) and mixed with 1 μg antibody or mouse/rabbit IgG followed by rotation at 4 °C overnight. Then, protein A or G Mag Sepharose was added into the mixture for further rotation at room temperature for 2 h. Sample was washed on Magnetic Rock 6 with PBS containing 0.5% NP‐40 for three times. After wash, the pellet was resuspended with 20 μL of 2 × sample buffer and denatured at 95 °C for 5 min followed by western blotting.

### 2.5 Flow cytometry

SkBr3 cell was treated with 10 μm U0126 for 6 h followed by stimulation with EGF for the indicated time. After treatment, cells were washed with PBS and trypsinized. Cells were double‐stained with anti‐HER2 antibody‐conjugated FITC and anti‐EGFR antibody‐conjugated PE at room temperature for 1 h, and subjected to flow cytometry analysis.

### 2.6 *In vitro* kinase assay

Recombinant GST‐HER2 1001‐1256 fusion protein and ERK kinase were mixed in kinase buffer (Cell Signaling #9802) containing 50 μm ATP and incubated at 30 °C for 30 min. The phosphorylation of GST‐HER2 at Thr701 by ERK was detected using western blot analysis with anti‐EGFR Thr669 antibody.

### 2.7 MTT assay

Cells were seeded at the density of 5 × 10^5^ cells/well in six‐well plate followed by RNA interference to knockdown clathrin. siRNA‐treated cells were re‐seeded at the density of 8 × 10^3^ cells/well in 96‐well plate. To measure the viability difference between the parental cells and clathrin‐knockdown cells after treatment with MEK inhibitors, the culture medium was removed and 80 μL of serum‐free medium and 20 μL of 5 mg·mL^−1^ MTT solution were mixed and added to each well followed by incubation at 37 °C for 3 h. Then, MTT solution was removed and 100 μL of DMSO was added to lyse the cells. After incubation for 1 h, the absorbance was detected by ELISA reader.

### 2.8 Proximity ligation assay (PLA)

Cells were seeded at the density of 1 × 10^5^ cells/slide and fixed with 4% paraformaldehyde for 10 min at room temperature followed by blocking with blocking solution (Duolink^®^ In Situ; Sigma, St. Louis, MO, USA) for 30 min at 37 °C. The slides were immunostained with anti‐EGFR‐ and anti‐HER2‐specific antibodies in a dilution of 1 : 100 at 4 °C overnight followed by the addition of PLA probe solution (Duolink^®^ In Situ; Sigma) and ligation ligase solution (Duolink^®^ In Situ; Sigma) for 1 h and 30 min, respectively. The signal was amplified by incubation with amplification polymerase solution (Duolink^®^ In Situ; Sigma) at 37 °C for 100 min. The visual spots at absorbance of 624 nm were observed by confocal microcopy.

### 2.9 Statistical analysis

Data were displayed as means ± SEM of three independent experimental replications. The significance of difference between the experimental and control groups was assessed by Student's *t*‐test. The difference is significant if the *P* value is < 0.05 (as noted as *) when compared to control group.

## 3 Results

### 3.1 MEK inhibitor induces Akt activation in a HER2‐dependent manner

**Figure 1:**
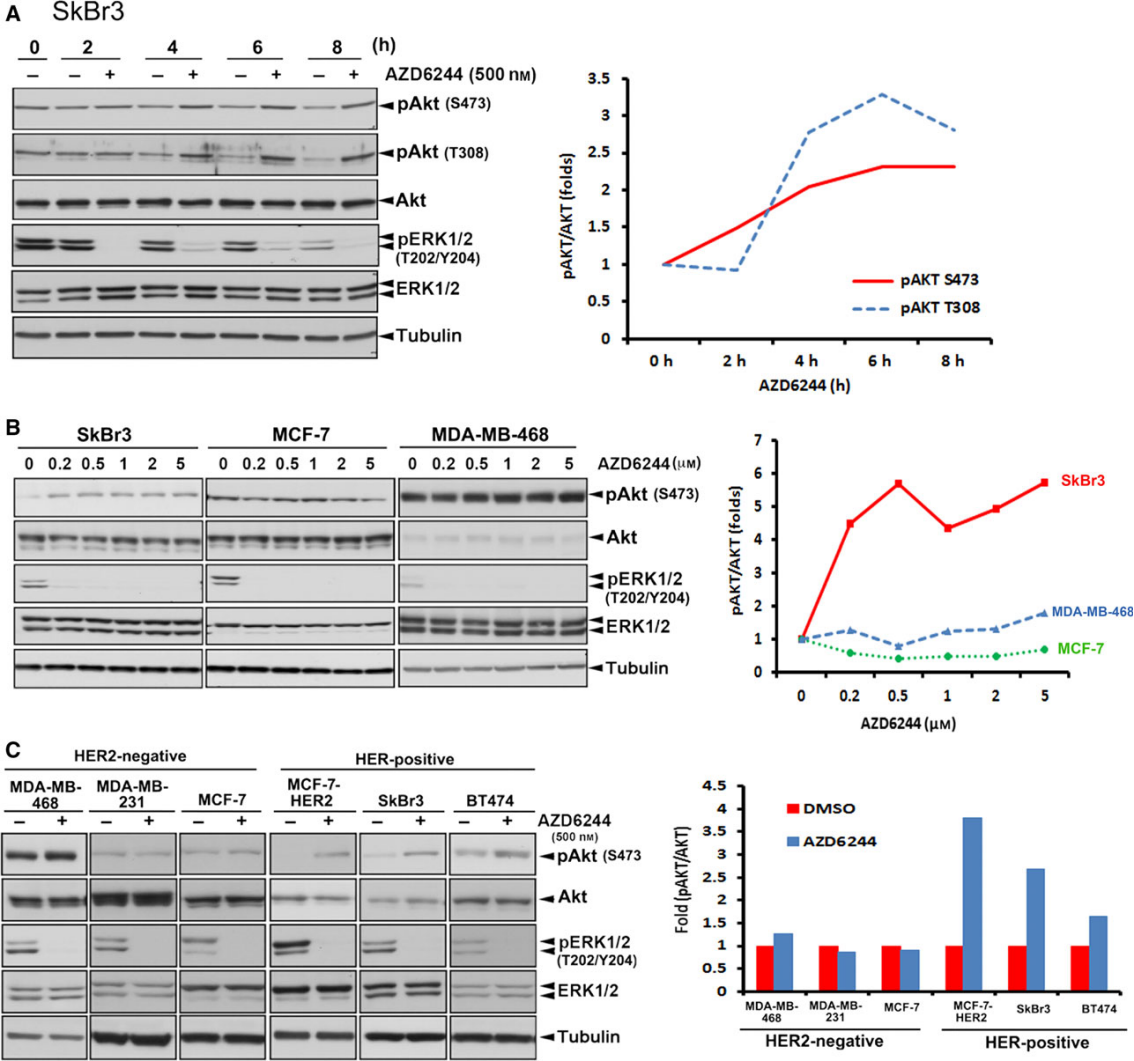
ERK inhibition induces Akt activations in HER2‐positive breast cancer cell lines. (A–C) Different breast cell lines were treated with 500 nm (A and C) or various concentrations (B) of AZD6244 for various time periods (A) or 6 h (B and C). Total protein lysates were then prepared and subjected to western blot analysis with indicated antibody. The changes in Akt phosphorylation normalized to total Akt protein were quantitated and shown at the right of each panel.

The activating phosphorylations of Akt at Ser473 and Thr308 were both induced by MEK inhibitor AZD6244 after four hours of treatment and reached the maximum after six hours in HER2‐positive SkBr3 breast cancer cell line (Fig. 1A). Treatment with AZD6244 also induced Akt Ser473 phosphorylation in SkBr3 cells in a dose‐dependent manner (Fig. 1B, left). However, the induction of Akt phosphorylation by AZD6244 was not observed in HER2‐negative MCF‐7 and MDA‐MB‐468 cells even when the treatment is up to 5 μm for 6 h (Fig. 1B, right). To further study whether HER2 plays a role in the induction of Akt signaling in response to ERK inhibition, we examined the inducing effect of AZD6244 on Akt Ser473 phosphorylation in different HER2‐positive and HER2‐negative breast cancer cell lines. As shown in Fig. 1C, AZD6244 induced Akt Ser473 phosphorylation only in HER2‐positive SkBr3 and BT474 but not in HER2‐negative MDA‐MB‐468, MDA‐MB‐231, and MCF‐7 breast cancer cell lines. But the induction of Akt phosphorylation by AZD6244 in MCF‐7 cells was found when HER2 was overexpressed (Fig. 1C). The activation of Akt by AZD6244 and U0126 was also found in two primary HER2‐positive cancer cells (Fig. S1). These results suggest that HER2 may play a critical role in the MEK inhibitor‐induced Akt activation.

**Figure 2:**
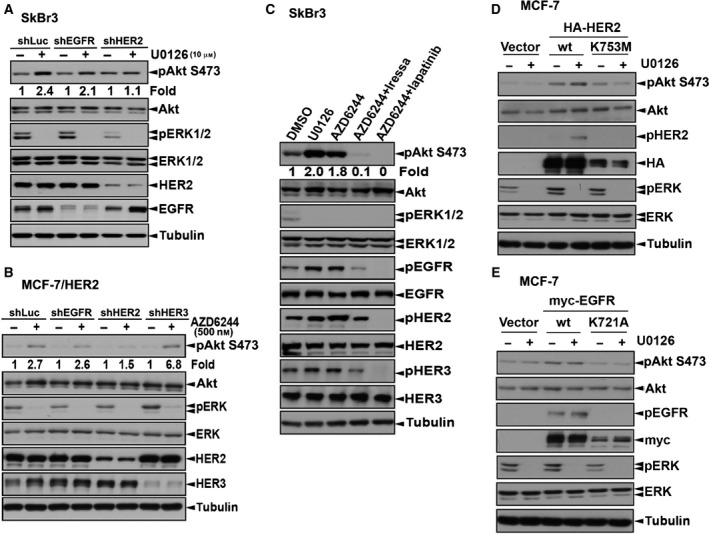
The tyrosine kinase activity of HER2 but not EGFR is sufficient for MEK inhibitor‐induced Akt activation. (A, B) SkBr3 cells (A) or HER2‐stable transfectant of MCF‐7 cells (B) were transfected with shEGFR and shHER2 plasmid followed by treatments with 10 μm U0126 or 500 nm AZD6244 for 6 h, respectively. (C) SkBr3 cells were treated with indicated inhibitors for 6 h. (D, E) MCF‐7 cells were transfected with HER2 (D), EGFR (E), or their kinase‐dead mutants followed by treatment with 10 μm U0126 for 6 h. Total lysates were prepared and subjected to western blot analysis with indicated antibodies.

To further demonstrate the necessity of HER2 in inducing Akt activity in response to ERK inhibition, the expressions of ErbB family were knocked down with shRNA in HER2‐positive cells for 72 h followed by treatment with MEK inhibitors for 6 h. As shown in Fig. 2A,B, silence of HER2 by shRNA dramatically attenuated the U0126‐ and AZD6244‐induced Akt phosphorylation in SkBr3 and MCF‐7/HER2 breast cancer cells, respectively, and EGFR shRNA showed slightly inhibitory effect on Akt activation in these cell lines; however, silence of HER3 did not show this inhibitory effect (Fig. 2B). Interestingly, both AZD6244 and U0126 can induce not only Akt activation but also the activating phosphorylations of EGFR at Tyr1068 and HER2 at Tyr1221/1222 (Fig. 2C). To further confirm that tyrosine kinase activity of EGFR/HER2 is required for the MEK inhibitor‐induced AKT phosphorylation, SkBr3 cells were treated with specific EGFR inhibitor gefitinib or dual EGFR/HER2 inhibitor lapatinib for 1 h after the treatment with AZD6244 for 6 h (Fig. 2C). Both gefitinib and lapatinib suppressed AZD6244‐induced kinase activations of EGFR, HER2, and Akt. Moreover, AZD6244 induced these phosphorylations of ErbB receptors and Akt in MCF‐7 cells transfected with wild‐type (wt) HER2 (Fig. 2D) but not EGFR (Fig. 2E) and their kinase‐dead (KD) mutants (Fig. 2D,E). These results indicated an important role of HER2 tyrosine kinase activity in MEK inhibitor‐induced Akt activation. Nevertheless, EGFR may be required but not sufficient to mediate this phenomenon.

### 3.2 Inhibition of HER2 Thr701 phosphorylation is critical for MEK inhibitor‐induced ErbB receptors and Akt activation

**Figure 3:**
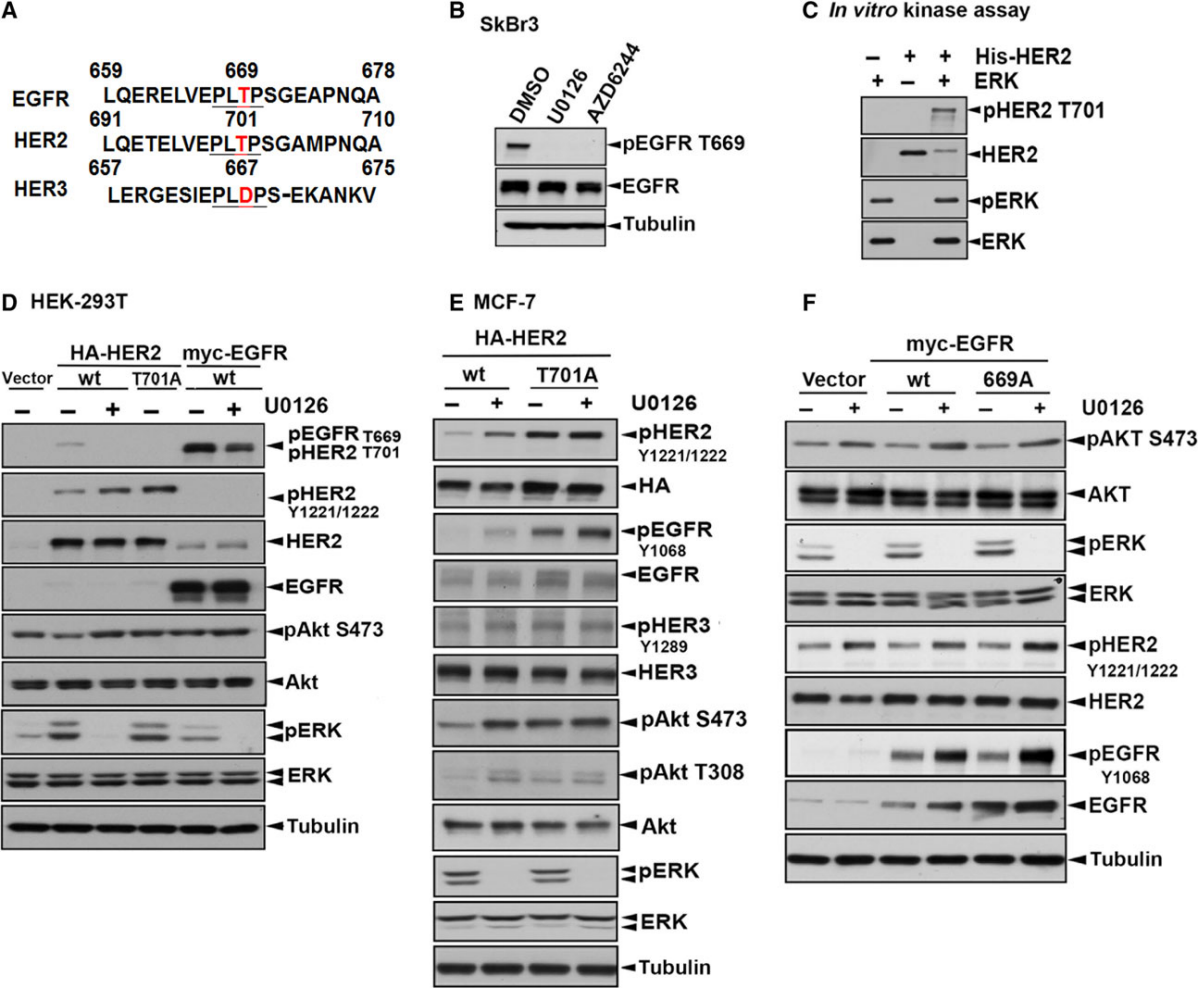
HER2 Thr701 phosphorylation by ERK mediates MEK inhibitor‐induced Akt activation. (A) Sequence alignment of EGFR, HER2, and HER3 at juxtamembrane regions that contain potential phosphorylation sites by ERK. (B) SkBr3 cells were treated with 10 μm U0126 or 500 nm AZD6244 for 6 h, and the EGFR phosphorylations at Thr669 were examined in western blot analysis. (C) Recombinant GST‐HER2 1001–1256 fusion protein and ERK kinase were mixed in kinase buffer and incubated at 30 °C for 30 min. The phosphorylation of GST‐HER2 at Thr701 by ERK was detected in western blot analysis with anti‐EGFR Thr669 antibody. (D–G) HEK‐293T (D) and MCF‐7 (E and F) cells were transfected with HA‐HER2, myc‐EGFR, and/or their mutants at Thr669 or Thr701 for ERK phosphorylations followed by treatment with U0126 for 6 h. The total lysates were prepared and subjected to western blot analysis with indicated antibodies.

We next addressed how MEK inhibitors induce Akt activation through ErbB receptor activation. MAPK has been reported to phosphorylate EGFR at Thr669 (Zwang and Yarden, 2006), which is conserved in HER2 at Thr701 but not in HER3 (Fig. 3A). Indeed, U0126 and AZD6244 can suppress EGFR Thr669 phosphorylation in SkBr3 cells, supporting that ERK phosphorylates EGFR at this residue (Fig. 3B). To examine whether ERK also phosphorylates HER2 at the conserved residue Thr701, we used recombinant ERK and his‐tagged HER2 as the kinase and the substrate, respectively, in the *in vitro* kinase assay followed by immunoblotting with anti‐EGFR Thr669 antibody. As shown in Fig. 3C, the phosphorylation of HER2 at Thr701, cross‐recognized by the anti‐EGFR Thr669 antibody (Cell Signaling #8808), was obviously induced in the presence of ERK. To further confirm this result, we transfected HA‐HER2 or myc‐EGFR into HEK‐293 cells, which do not express these ErbB receptors. The phosphorylations of EGFR at Thr669 and HER2 at Thr701 were both detected by this antibody and inhibited by AZD6244. The substitution of Thr701 to Ala also inhibited this ERK‐mediated HER2 phosphorylation (Fig. 3D). These results indicate that ERK also phosphorylates HER2 at the conserved residues Thr701.

Like the treatment with MEK inhibitors, the unphosphorylation mimicking mutation of HER2 Thr701 to Ala (HER2 T701A) also increased Akt Ser437 and Thr308 phosphorylations as well as the activating phosphorylations of HER2 at Tyr1221/1222 and EGFR at Tyr1068 but not HER3 at Tyr1289 in HER2‐transfected HEK‐293T and MCF‐7 cells (Fig. 3D,E). More importantly, these phosphorylations were not further enhanced by U0126 in the presence of HER2 Thr701 mutant (Fig. 3E). However, the unphosphorylation mimicking mutation of EGFR Thr669 to Ala (EGFR T669A) did not increase Akt Ser473, EGFR Tyr1068, and HER2 Tyr1221/122 phosphorylations. The U0126‐induced EGFR and HER2 tyrosine phosphorylations were also not affected by the EGFR Thr669 mutation (Fig. 3F). These results suggest that inhibition of HER2 Thr701 but not EGFR Thr669 phosphorylation by MEK inhibitors can enhance the tyrosine kinase activity of HER2 to transactivate EGFR and its downstream Akt pathway although ERK can phosphorylate both EGFR and HER2 at these residues.

### 3.3 HER2 T701 phosphorylation by ERK causes feedback downregulation of HER2 and EGFR interaction

**Figure 4:**
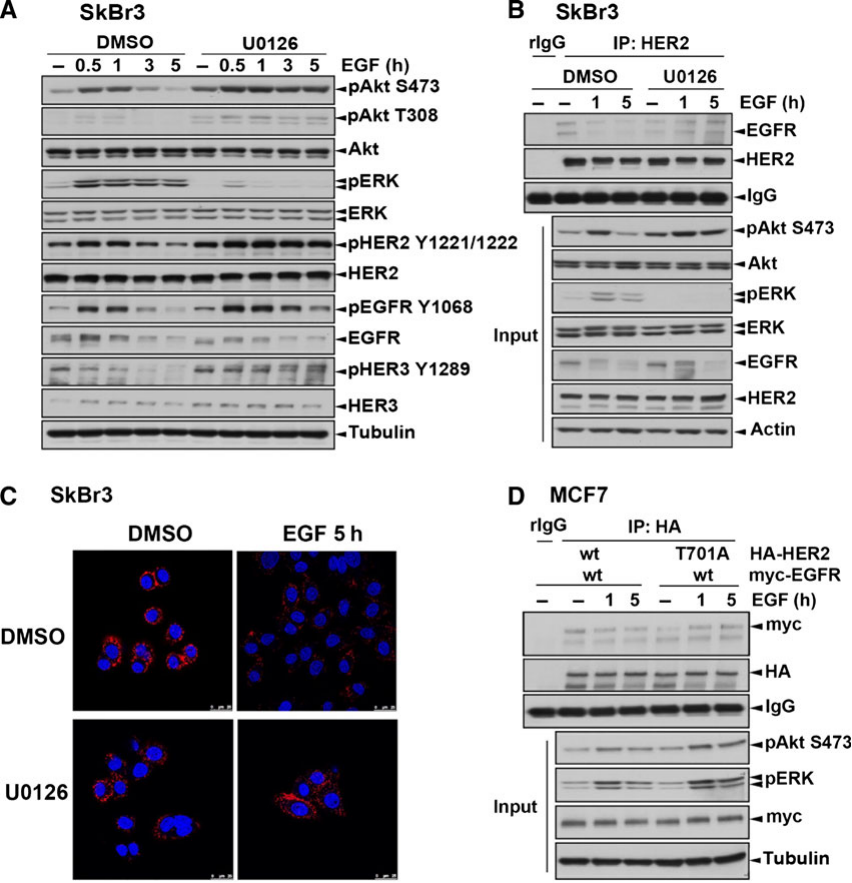
Suppression of HER2 Thr701 phosphorylation by MEK inhibitors increases HER2/EGFR heterodimerization. (A, B) SkBr3 cells were pretreated with U0126 for 2 h followed by stimulation with EGF for indicated time periods. Total lysates were prepared and subjected to direct western blot or anti‐HER2 immunoprecipitation/western blot analysis with indicated antibodies. (C) SkBr3 cells were pretreated with U0126 for 2 h followed by stimulations with EGF for 5 h. Cells were fixed and stained with anti‐EGFR and anti‐HER2 antibodies. The interaction between EGFR and HER2 is represented as red dots in PLA analysis. (D) MCF‐7 cells transfected with HA‐HER2, myc‐EGFR, and/or HA‐HER2 T701A mutant were stimulated with EGF for 1 or 5 h. Total lysates were prepared and subjected to anti‐HA immunoprecipitation/western blot analysis.

To further study whether HER2 T701 phosphorylation plays a role in feedback negatively regulating EGFR and HER2 kinase activity, we pretreated SkBr3 cells with MEK inhibitors followed by EGF stimulation. As shown in Fig. 4A, EGFR and HER2 kinase activity and their downstream Akt and ERK signaling were dramatically induced by EGF and achieved the maximum after 30 min, but were declined after three hours. However, pretreatment with U0126 obviously sustained HER2 and EGFR kinase activities and Akt signaling. We next addressed whether the prolongation of EGFR/HER2 activation and Akt signaling by U0126 is due to the enhancement of protein interaction between EGFR and HER2. To this end, SkBr3 cells were pretreated with U0126 for 2 h followed by EGF stimulation for various time points, and then total lysates were prepared for immunoprecipitation with anti‐HER2 antibody and subsequent immunoblot analysis with anti‐EGFR antibody. The interaction between EGFR and HER2 was induced after 15 min of treatment and sustained for 60 min, and this interaction was not affected by U0126 (Fig. S2). However, the interaction of HER2 with EGFR was declined after EGF stimulation for 5 h, but pretreatment with U0126 significantly enhanced this protein interaction (Fig. 4B). The enhancing effect of U0126 on the interaction between EGFR and HER2 in response to EGF stimulation was also observed in proximal ligation assay (PLA) (Fig. 4C). To further demonstrate that HER2 T701 phosphorylation mediates the feedback downregulation of EGFR/HER2 interaction in response to EGF stimulation, MCF‐7 cells were cotransfected with EGFR and HER2 for IP/WB analysis. Similar to MEK inhibitors, mutation of HER2 T701 to Ala also increased the EGF‐induced EGFR/HER2 interaction (Fig. 4D).

**Figure 5:**
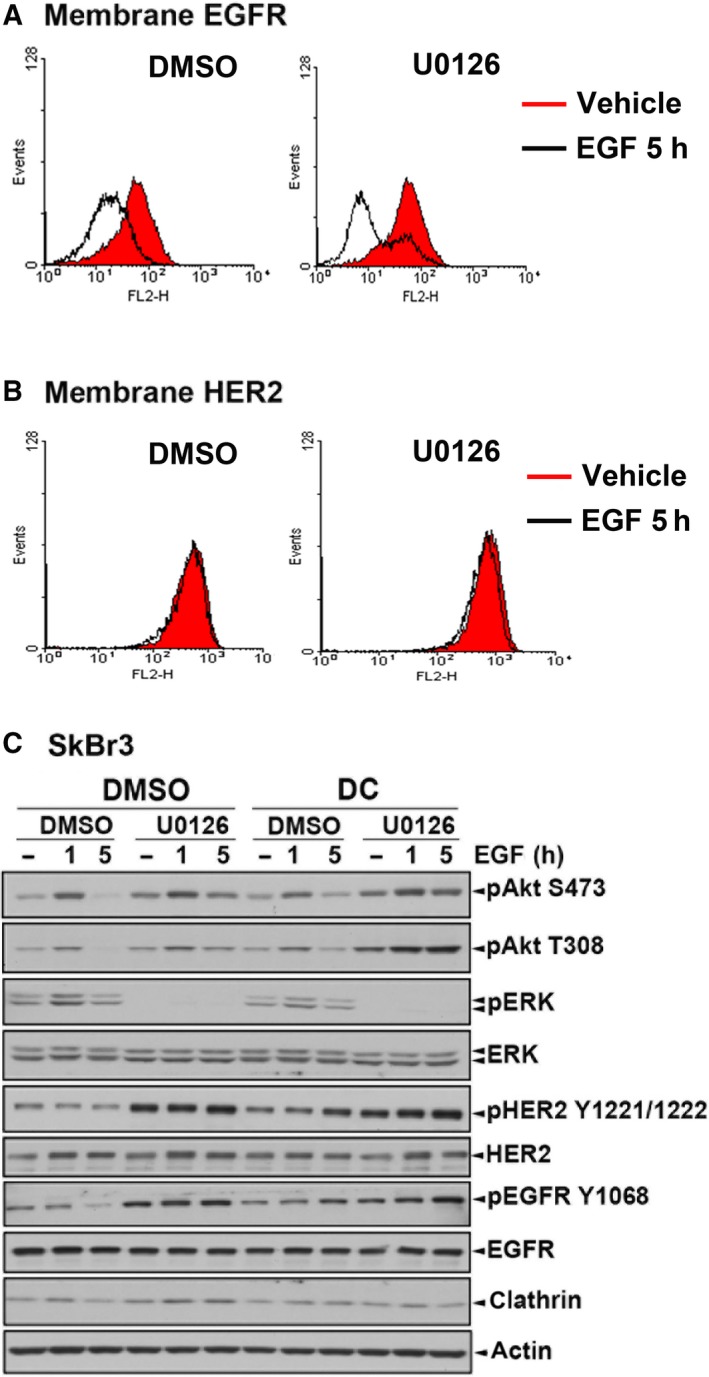
Reduction in EGF‐induced EGFR internalization by MEK inhibitors prolongs Akt activations. (A, B) SkBr3 cells were pretreated with U0126 followed by stimulation with EGF for 5 h and then stained with anti‐EGFR (A) or anti‐HER2 (B) antibodies. The membrane levels of EGFR (A) and HER2 (B) were measured in flow cytometry analysis. (C) SkBr3 cells were pretreated with U0126 and/or dansylcadaverine (DC) followed by stimulations with EGF for 1 or 5 h. Total protein lysates were prepared and indicated protein level and phosphorylations were determined in western blot analysis with specific antibodies.

**Figure 6:**
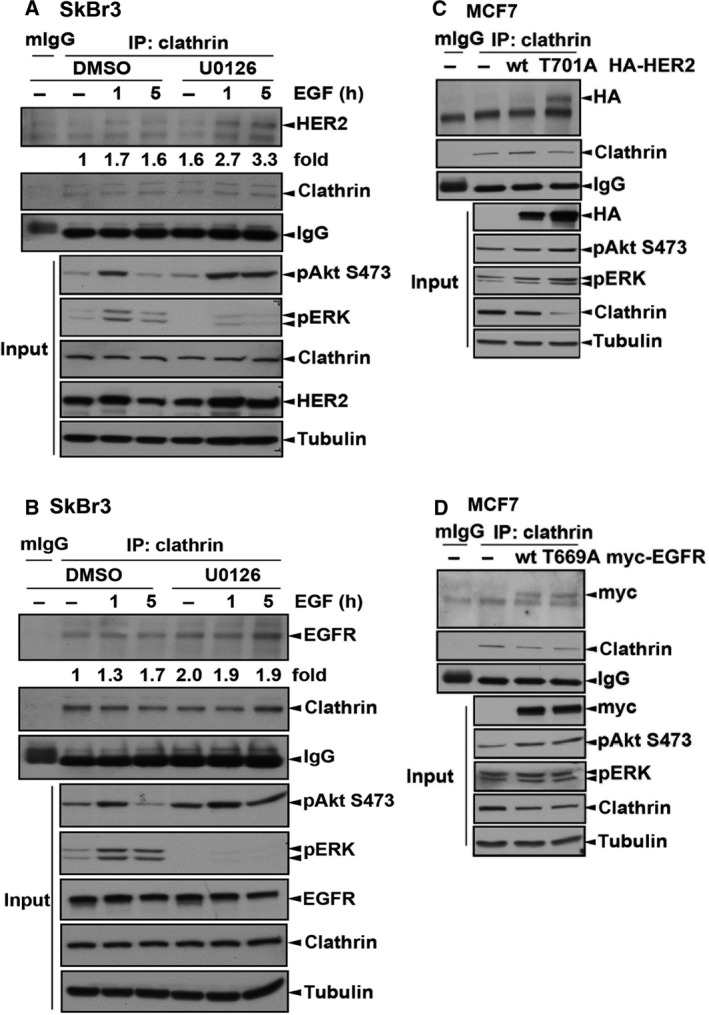
Loss of HER2 Thr701 phosphorylation increases the interaction between clathrin and HER2. (A, B) SkBr3 cells were pretreated with U0126 for 2 h followed by stimulation with EGF for indicated time periods. Total lysates were prepared and subjected to anti‐clathrin immunoprecipitation/western blot analysis with indicated antibodies. (C, D) MCF‐7 cells transfected with HA‐HER2 wt and its T701A mutant (C) or with myc‐EGFR wt or its T669A mutant (D) were stimulated with EGF for 1 or 5 h. Total lysates were prepared and subjected to anti‐clathrin immunoprecipitation/western blot analysis.

**Figure 7:**
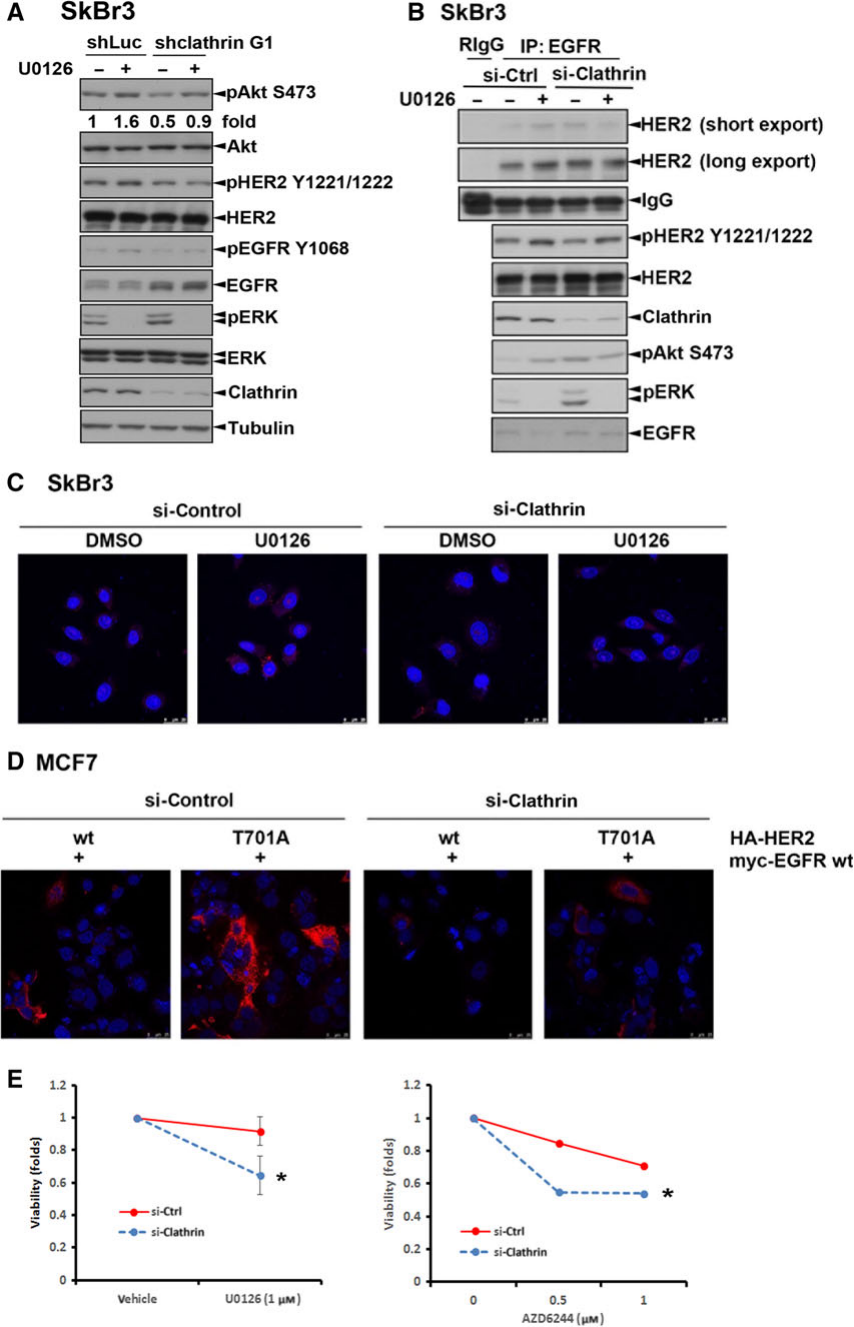
The increased interaction with clathrin enhances HER2/EGFR heterodimerization and Akt activity. (A, B) SkBr3 cells transfected with clathrin shRNA or siRNA were treated with U0126 for 6 h. Total lysates were prepared and subjected to direct western blot (A) or anti‐EGFR immunoprecipitation/western blot analysis with indicated antibodies. (C, D) SkBr3 cells were treated with U0126 for 6 h (C). MCF‐7 cells transfected with HA‐HER2, myc‐EGFR, and/or HA‐HER2 T701A mutant were cotransfected with control of clathrin siRNA for 2 days (D). These cells were then fixed and stained with anti‐EGFR and anti‐HER2 antibodies, and the interaction between EGFR and HER2 is represented as red dots in PLA analysis. (E) SkBr3 cells transfected with control or clathrin siRNA were treated with U0126 or AZD6244 for 48 h. The cell viability was determined by MTT analysis. Statistical analysis was performed by *t*‐test. *P < 0.05 as compared to control group.

The removal of RTK from the cell surface to endocytic internalization and degradation is the major pathway for RTK signaling attenuation. Therefore, we next examined whether ERK‐dependent HER2 T701 phosphorylation regulates the internalization of EGFR and HER2. Upon stimulation of EGF for 5 h, the cell surface EGFR but not HER2 expression detected in flow cytometry analysis was dramatically downregulated in SkBr3 cells. However, pretreatment with U0126 for 2 h can slow down the EGF‐induced internalization of EGFR (Fig. 5A) without affecting the surface amount of HER2 (Fig. 5B). Pretreatment with internalization inhibitor dansylcadaverine (DC) also enhanced U0126‐induced EGFR/HER2 and Akt activations (Fig. 5C), implying that inhibition of ERK prolongs the duration of EGFR/HER2 signaling by retaining these receptors on the cell surface membrane. On the cell surface, ligand/receptor complexes can be further recruited into clathrin‐coated pits (CCP) and can invaginate inward and pinch off vesicles into the cytoplasm. Clathrin‐mediated endocytosis (CME) mainly mediates the internalization of EGFR and HER2 and sustains the duration of EGFR/HER2 signaling by skewing the fate of internalized RTK toward recycling rather than degradation (Garay *et al*., 2015; Sigismund *et al*., 2008). After stimulation with EGF for 1 or 5 h, the interaction of clathrin with HER2 (Fig. 6A) but not EGFR (Fig. 6B) was dramatically increased by pretreatment with U0126. Mutation of HER2 T701 to Ala also obviously increased the protein–protein binding activity of HER2 and clathrin (Fig. 6C). However, mutation of EGFR T669 to Ala did not show the similar result (Fig. 6D). When clathrin was knocked down by specific shRNA, U0126‐induced HER2, EGFR and Akt activations were attenuated (Fig. 7A). The U0126‐induced heterodimerization between HER2 and EGFR was also blocked by clathrin siRNA (Fig. 7B). To further support the involvement of clathrin in the U0126‐induced EGFR/HER2 dimerization, silence of clathrin also suppressed U0126‐induced complex formation of EGFR and HER2 in PLA (Fig. 7C). Mutation of HER2 T701 to Ala also enhanced the protein–protein interaction between HER2 and EGFR, and this effect was also inhibited by clathrin siRNA (Fig. 7D). Silence of clathrin also enhanced U0126‐ or AZD6244‐induced cytotoxicity of SkBr3 cells in MTT assay (Fig. 7E).

**Figure 8:**
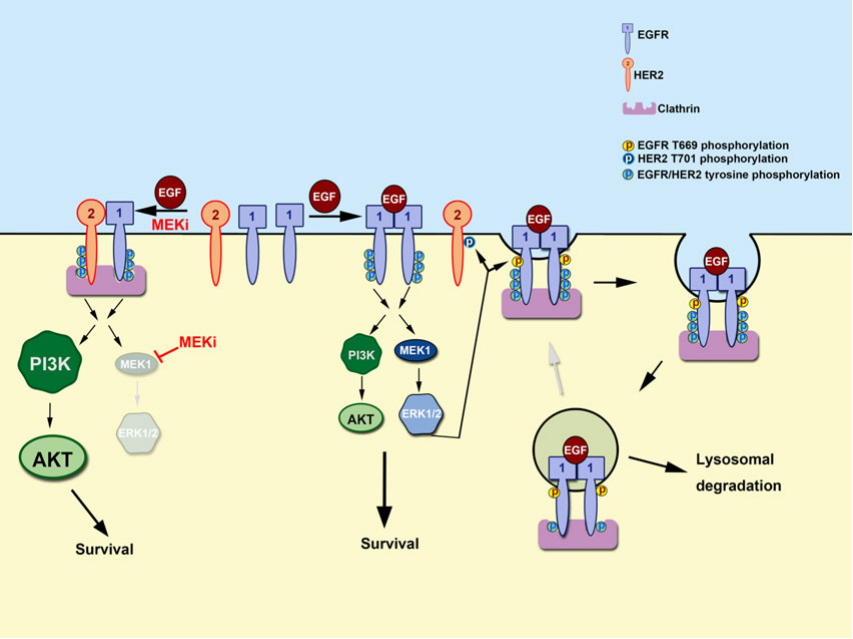
MEK inhibitors suppress ERK‐mediated HER2 Thr701 phosphorylation to enhance clathrin‐dependent HER2/EGFR dimerization and subsequent Akt activation. ERK1/2 activated by RTK/MEK signaling axis feedback phosphorylates HER2 and EGFR at Thr701 and Thr669, respectively, in response to EGF stimulation. HER2 Thr701 phosphorylation negatively regulates the heterodimerization of EGFR and HER2 via reducing the clathrin‐binding activity of HER2, resulting in the clathrin‐mediated endocytosis of EGFR homodimer to attenuate EGF signaling. Suppression of ERK activity by MEK inhibitors relieves the negative regulation on the clathrin‐dependent heterodimerization of HER2 and EGFR by reducing HER2 Thr701 phosphorylation. The increased binding between EGFR and HER2 enhanced and prolonged the activation of these RTKs and the downstream PI3K/Akt pathway, and may thereby contribute to the drug resistance to MEK inhibitors.

Taken together, ERK feedback phosphorylates HER2 at Thr701 to negatively regulate clathrin‐mediated EGFR/HER2 interaction and endocytic recycling, and thereby contributes to the termination of EGF signaling. Therefore, reduction in this phosphorylation by inhibiting MEK enhances EGFR/HER2 signaling and Akt activation, accounting for the plausible mechanisms of drug resistance to ERK inhibitors (Fig. 8).

## 4 Discussion

In addition to driving signaling intermediates, including Akt, MAPK, and PLC pathways for the regulations of cell survival and metabolism, activated EGFR RTK family also initiates early and delayed regulatory loops to dictate the duration and amplitude of signal after binding with ligands. Unlike the involvement of gene transcription in the delayed regulatory loop, the early events compass both protein translocations and post‐translational modifications, including the ligand‐induced receptor endocytosis, ubiquitination, and phosphorylations (Avraham and Yarden, 2011). In this study, we discovered a cross‐talk between threonine phosphorylation and CME in the negative feedback regulation of EGFR/HER2 heterodimer.

Concomitant to the activation of signaling pathway, ligand‐bound EGFR complex undergoes internalization through CME at low dose of EGF stimulation or clathrin‐independent endocytosis at high ligand concentrations (Sigismund *et al*., 2008). As a scaffold protein, clathrin recruits the cargo protein EGFR and many additional cytosolic proteins, and assembles them into CCP. After membrane ingression, the GTPase dynamin processes the scission of CCPs from plasma membrane to form intracellular clathrin‐coated vesicles for delivering EGFR to endosomes. In the endosomes, EGFR can then be either tagged for degradation or recycle back to the plasma membrane, which both occurs about 30 min to hour after ligand stimulation (Sorkin and Goh, 2009). Although endocytosis is a major negative feedback loop for EGFR, certain downstream signaling pathways still can be driven from the internalized receptors in endosomes while on the route for recycling or degradation. Clathrin‐coated membrane microdomains have been proposed to be particularly important for the activation of AKT and MAPK upon EGFR activation (Kim *et al*., 2002). Nevertheless, EGFR transferred from CCP at plasma membrane to endosomes terminates PI3K–AKT signaling, while prolonging MAPK signaling (Fehrenbacher *et al*., 2009; Vieira *et al*., 1996). EGFR preferentially resides within a clathrin‐coated microenvironment at the plasma membrane to activate Gab1‐PI3K‐Akt pathway. Silence of clathrin, but not dynamin, was found to attenuate EGF‐stimulated Akt activation, indicating that clathrin regulates EGFR signaling at the plasma membrane (Garay *et al*., 2015). Our data showing EGF‐induced Akt activation in the presence of endocytosis inhibitor DC also support this notion. However, it was unclear whether the prolonged MAPK pathway is involved in the termination of clathrin‐mediated Akt signaling.

The binding with ligands leads to both EGFR homodimerization and heterodimerization with members of the HER kinase family. Feedback phosphorylation of EGFR at Thr669 by ERK has been found to negatively regulate constitutive EGFR tyrosine kinase activity and HER3‐driven Akt activation by suppressing heterodimerization of EGFR/HER3 in lung cancer and triple‐negative MDA‐MB‐468 breast cancer cells (Sato *et al*., 2013; Turke *et al*., 2012). In response to cisplatin treatment, phosphorylation of EGFR at Thr669 is mediated by p38 but not ERK, and contributes to EGFR internalization in triple‐negative MDA‐MB‐468 cells. However, Akt phosphorylation is upregulated but not downregulated in the cisplatin‐treated cells (Winograd‐Katz and Levitzki, 2006). These conflict findings suggest that the role of phosphorylation of EGFR at Thr669 in determining the status of Akt phosphorylation remains controversial. Although phosphorylation of this threonine residue has been shown to impair EGFR activation, likely through interfering receptor dimerization (Li *et al*., 2008), the underlying molecular mechanism is not unclear. In this study, we identified HER2 Thr701 also as a phosphorylation target for ERK, and this conserved phosphorylation site was not found in HER3. Consistently, our data also showed that HER2, but not HER3, is involved in the heterodimerization with EGFR and in the regulation of EGF‐induced Akt signaling in HER2‐positive breast cancer cells. In support of our findings, the dominant role of HER2 but not HER3 in the ERK‐dependent EGFR internalization and Akt downregulation was found in prostate cancer and retinal pigment epithelial cells (Gao *et al*., 2012; Garay *et al*., 2015). These results suggest that the involvement of other members of HER kinase family in the ERK‐dependent feedback downregulation of EGFR and Akt signaling may be context specific (Hendriks *et al*., 2003).

Our results indicate that clathrin plays a critical regulator in the ERK‐mediated negative feedback regulation of HER2/EGFR activity and Akt activity. Although HER2 internalization was found in geldanamycin‐treated cells and is a clathrin‐dependent process (Lerdrup *et al*., 2007; Pedersen *et al*., 2008), many studies indicated that HER2‐containing heterodimers is internalization resistant in response to ligand stimulation (Lenferink *et al*., 1998; Worthylake *et al*., 1999) probably due to the lack of an internalization motif in the cytoplasmic domain of HER2 (Sorkin *et al*., 1993). Moreover, HER2 inhibits EGF‐induced EGFR internalization by forming EGFR/ErbB2 heterodimerization without affecting the phosphorylation or ubiquitination of EGFR (Haslekas *et al*., 2005; Wang *et al*., 1999), and by having a negative effect on the formation of CCP (Cortese *et al*., 2013). Consistently, our data showed that EGF induced downregulation of membrane EGFR but not HER2 level. Interestingly, the downregulation of membrane EGFR by EGF stimulation was reversed in the presence of MEK inhibitor (Fig. 5A,B). Inhibition of HER2 T701 phosphorylation by MEK inhibitor or by mutation of this site dramatically prolongs the formation of EGFR/HER2 heterodimer (Fig. 4) and increased the interaction of HER2 with clathrin (Fig. 5). However, mutation of EGFR Thr669 did not show the similar effect. Therefore, our data suggest that the reduction in HER2 Thr701 phosphorylation by ERK inhibition may specifically inhibit CME of EGFR through increasing the clathrin binding and sequestering by HER2, and thereby enhances the retention of EGFR at plasma membrane for the dimerization with HER2, explaining the sustained and augmented Akt activity in response to the treatment with MEK inhibitor.

In this study, our data showed that the loss of HER2 Thr701 increases the interaction between HER2 and clathrin to enhance the formation of EGFR/HER2 heterodimer and Akt activation, conferring the resistance to MEK inhibitor. Silence of clathrin by siRNA thereby can sensitize HER2‐positive breast cancer cells to AZD6244. Combination therapy with MEK inhibitor trametinib and EGFR/HER2 dual inhibitor lapatinib has shown a synergistic effect and circumvented the resistance of MEK1‐mutated gastric cancer cells (Mizukami *et al*., 2015) and HER2‐positive breast cancer cells (Karakashev and Reginato, 2015) to MEK inhibitors. Furthermore, cotreatment with EGFR monoclonal antibody panitumumab and HER2 monoclonal antibody trastuzumab also augments the response of patient‐derived xenograft pancreatic tumor to MEK inhibitor trametinib (Lindberg *et al*., 2014). In these studies, it was commonly found that HER2/EGFR/Akt signaling axis was activated by trametinib and that cotreatment with HER2 inhibitors synergizes the anticancer activity of trametinib in various cancer types. Our findings that MEK inhibitors relieve the feedback inhibitory phosphorylations of HER2 at Thr701 by ERK for the compensatory Akt activation in a clathrin‐dependent manner could further explain this synergism. Notably, HER2 is less expressed in melanoma (Kluger *et al*., 2004), which is commonly sensitive to MEK inhibitor. It would be interesting to further study whether HER2 expression or HER2 T701 phosphorylation is a critical biomarker for the prediction of the antitumor efficiency of MEK inhibitors in melanoma cells or other cancer types.

## 5 Conclusion

In this study, our data showed that HER2 is essential for the compensatory Akt activation in response to MEK inhibitors. ERK phosphorylates HER2 at Thr701, and inhibition of HER2 Thr701 phosphorylation by MEK inhibitors increases HER2/EGFR interaction via binding to clathrin for the compensatory Akt activation and resistance to MEK inhibitors. These findings not only decipher the essential role of clathrin in the feedback EGFR/HER2 and Akt activation in response to ERK inhibition by MEK inhibitor, but also suggest HER2 expression as a potential marker to predict the therapeutic efficacy of patients with cancer to MEK inhibitors.

## Author contributions

WCH and CYT involved in conception of study. WCH and CYT designed the study. CHC, TCH, MHY, and YLW acquired the data. CHC and YLW involved in quality control of data and algorithms. CHC and JTC analyzed and interpreted the data. CHC and YJC involved in statistical analysis. WCH, CHC, and CYT prepared the manuscript. CYT involved in manuscript editing. WCH reviewed the manuscript. All authors read and approved the final manuscript.

## Supporting information


**Fig. S1.** MEK inhibitors induce Akt activation in primary HER2‐positive breast cancer cells.
**Fig. S2.** MEK inhibitor did not affect the initiation of EGF‐induced EGFR/HER2 interaction.
**Fig. S3.** The raw data of Figs 1c and 2a.
