# Supplementary material for: MEK inhibitors induce Akt activation and drug resistance by suppressing negative feedback ERK‐mediated HER2 phosphorylation at Thr701
Source: Mol Oncol. 2017 Jul 19;11(9):1273–87. doi: 10.1002/1878-0261.12102 (PMC5579385; doi:10.1002/1878-0261.12102)
Supplement: Supplementary file 1 — Fig. S1. MEK inhibitors induce Akt activation in primary HER2‐positive breast cancer cells. Fig. S2. MEK inhibitor did not affect the initiation of EGF‐induced EGFR/HER2 interaction. Fig. S3. The raw data of Figs 1c and 2a. [file MOL2-11-1273-s001.pdf]

Supplementary Figures

Supplementary Figure S1.

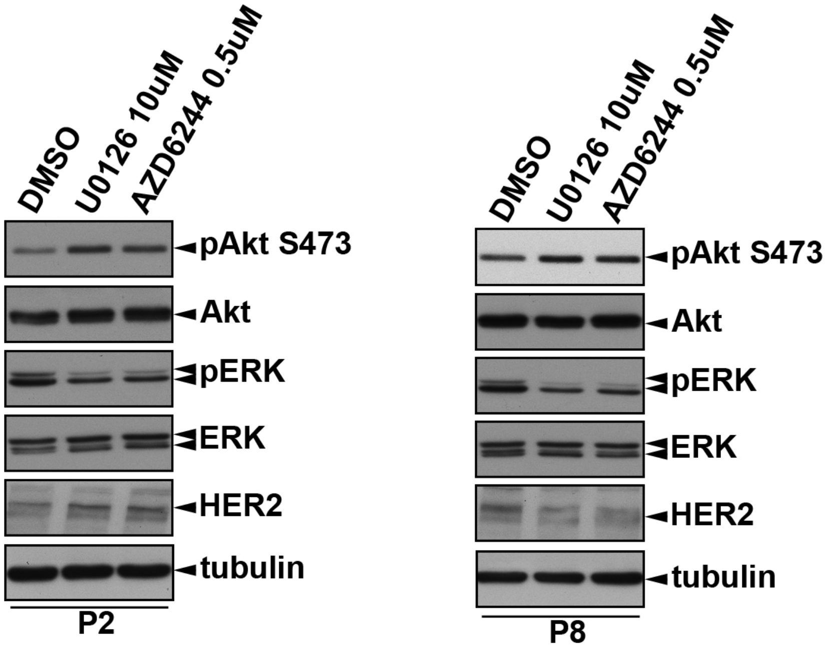

Supplementary Figure S2.

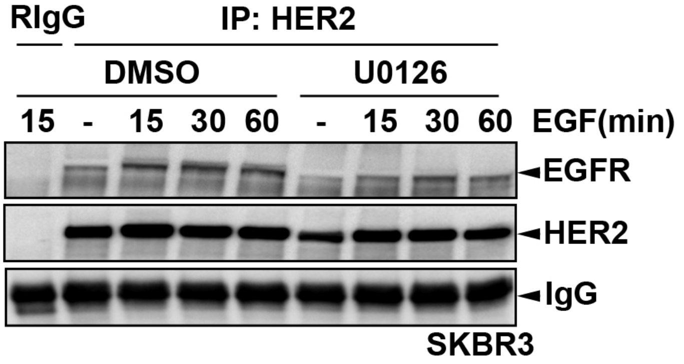

Supplementary Figure S3.

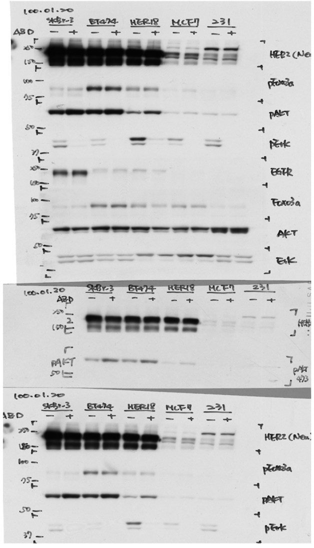
HER2
pAKT
pErk
AKT
Erk

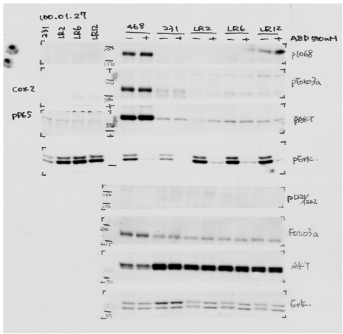
AKT

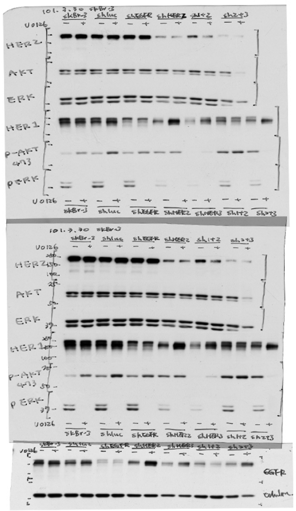
HER2
AKT
ERK
